# Application of serial tests for *Mycobacterium tuberculosis* detection to active lung tuberculosis cases in Indonesia

**DOI:** 10.1186/s13104-019-4350-9

**Published:** 2019-06-03

**Authors:** Nastiti Intan Permata Sari, Ni Made Mertaniasih, Soedarsono, Fumito Maruyama

**Affiliations:** 1 Doctoral Program of Medical Science, Faculty of Medicine, Universitas Airlangga, Jl. Mayjen. Prof. Dr. Moestopo No. 47, Surabaya, 60131 Indonesia; 2 Laboratory of Tuberculosis, Institute of Tropical Disease, Universitas Airlangga, Kampus C, Jl. Mulyorejo, Surabaya, 60115 Indonesia; 3 Department of Clinical Microbiology, Faculty of Medicine, Universitas Airlangga, Dr. Soetomo Hospital, Jl. Mayjen. Prof. Dr. Moestopo No. 47, Surabaya, 60131 Indonesia; 4 Department of Pulmonology and Respiratory Medicine, Faculty of Medicine, Universitas Airlangga, Jl. Mayjen. Prof. Dr. Moestopo No. 47, Surabaya, 60131 Indonesia; 5 Department of Microbiology, Graduate School of Medicine, Kyoto University, Kyoto, Japan

**Keywords:** Acid fast bacilli, Gene Xpert, Culture method, PCR method, Pulmonary tuberculosis, Serial tests

## Abstract

**Objective:**

Rapid detection and accurate diagnosis are very important in managing active tuberculosis because they provide an advantage in preventing further disease transmission. In accordance with the recommendation of the World Health Organization, the Indonesian Tuberculosis Control Program uses the acid fast bacilli (AFB) smear and Chest X-ray methods as the primary methods for detecting tuberculosis, especially in new cases of suspected tuberculosis. The genus *Mycobacterium* has many species, strains, and variants, and their natural differences may affect the clinical outcome of the diseases they induce. The purpose of this study was to assess different tuberculosis detection methods as part of serial tests and determine the best diagnostic approach for detecting active lung tuberculosis in Indonesia.

**Results:**

This study used clinical samples from tuberculosis patients and assessed them using a series of tests, aiming to increase the sensitivity of active tuberculosis detection. Some samples that yielded negative results in the AFB smear test were detected as positive for *Mycobacterium tuberculosis* using the nucleic acid amplification test, with a sensitivity of 83.1%. Additionally, nucleic acid amplification also detected positive results among samples assessed as *M. tuberculosis*-negative using the culture method, this method yielded the same results as the Gene Xpert test.

## Introduction

Tuberculosis (TB) is a major global health problem; this disease has caused more deaths than any other infectious disease over the last 200 years [1]. The number of TB deaths remains unacceptably high. To combat this, a timely diagnosis and the application of appropriate treatment are required [2]. TB infection is caused by members of the *Mycobacterium tuberculosis* Complex (MTBC), which is the major group responsible for human pulmonary infections. This group is composed of anaerobic, acid-fast, slow-growing bacteria, and it can be difficult to determine the species responsible for specific TB cases [3]. TB usually appears several years after infection, often when the immune system function is decreased by some other cause [4]. The transmission of TB occurs by inhalation of infectious droplet nuclei containing viable bacilli (aerosol spread). Factors influencing the chance of transmission include the bacillary load of the source case, e.g., higher transmission occurs from active pulmonary cases who are sputum smear-positive or who display lung cavities on a chest radiograph, as well as the proximity and duration of exposure [5]. Overall, the risk of infection among household contacts of TB patients is around 30% [6]. Transmission is rapidly reduced with effective treatment [7]. Therefore, early TB diagnosis and management is critical.

An acid-fast bacilli (AFB) smear is used to screen for new TB infection, and this method requires fresh sputum from patients as input samples. Previous work in Brazil on the accuracy of the AFB smear method reported that this test has a sensitivity of 36% and a specificity of 100% [8].

The conventional culture method for *Mycobacterium tuberculosis* is a more sensitive technique by which to detect TB compared with AFB smears, but, due to the slow growth of the organism, sputum cultures take 4–6 weeks to become positive on solid media and 10–21 days in liquid media. Solid culture is usually performed on Lowenstein–Jensen (LJ), Ogawa, or Middlebrook 7H10/11. Liquid culture of *M. tuberculosis* is more sensitive and rapid than solid culture but is prone to contamination in some laboratories, so it is best to use both methods in conjunction [9].

The significant advance in the diagnosis of TB in the last decade has been the advent of the Gene Xpert MTB/RIF (Gene Xpert) test. The accuracy of the Gene Xpert test offers a significant increase in terms of diagnostic sensitivity, even when it is deployed selectively, i.e., among only smear-negative presumptive TB patients. However, because this method is limited by the expensive equipment required, the Gene Xpert assay is still rarely used in countries with a high HIV/TB prevalence [10].

The detection of *M. tuberculosis* in clinical samples is generally less sensitive than the detection of other pathogens due to the relatively low numbers of bacilli present [11]. Despite this limitation, the nucleic acid amplification method has the potential to be used for differentiating the species and strains of isolates and clinical specimens [12].

In Indonesia, new patient TB is initially diagnosed by the AFB smear and X-ray methods. Following positive results from these tests, the patient status is further monitored by the culture method during the treatment period. Traditional TB control focused on the identification and treatment of sputum smear-positive TB patients, who are considered the most infectious cases. In a policy change in 2010, the World Health Organization recommended that two sputum samples are sufficient, rather than the standard three samples (spot-morning-spot) that had been recommended for several decades [13]. Repeated testing may still be warranted, so it is important to establish a good diagnostic method for detecting active lung TB.

The methods for MTBC detection have variable accuracy, and a combination of serial tests is expected to increase the overall accuracy of the diagnostic method. Therefore, this study investigated the diagnostic quality of applying serial MTBC detection tests to detecting active lung TB in Indonesia.

## Main text

### Methods

This study was conducted on sputum samples from pulmonary TB patients. All samples were examined by serial tests: smear microscopy, culture method, Gene Xpert assay, and PCR assay. All methods were used to evaluate the sensitivity and specificity for detecting active TB.

#### Patients

From September to December 2016, sputum samples were collected 96 samples by random sampling which collected from minimum representative samples of pulmonary active TB patients with positive and negative AFB results, who were either untreated or treated with anti-TB drugs, in the Dr. Soetomo Academic Hospital, Surabaya, Indonesia. These samples were then examined using smear microscopy, culture, PCR, and the Gene Xpert assay. The Gene Xpert assay was used for the screening of antibiotic-resistant cases. For each patient, the sex (male or female), age group (< 30 years old; 30–60 years old; or > 60 years old), and length of drug treatment (< 6 months or > 6 months) were recorded.

#### Smear microscopy

The sputum samples were first decontaminated with 4% NaOH as previously described [14]. After decontamination, a circular smear of each sputum sample was made on slides with a 2 × 3-cm^2^ surface area. For the AFB smears, the Ziehl-Neelsen staining method was used as previously described [15, 16].

#### Mycobacterial culture

The culture method was performed using Lowenstein-Jensen and Middlebrook media (Merck, Germany) [17]. This method requires around 3–4 weeks for visible colonies to grow. After the colonies appeared, they were subjected to the SD Bioline MPT64 antigen test (Standard Diagnostic, Germany) to identify the MTBC species.

#### DNA extraction

DNA extraction was conducted using the Qiagen DNA extraction kit (DNeasy^®^, Cat. No. 69504). Extracted DNA was amplified using PCR (MJ MiniTM Thermal cycle, BioRed). The primers used were F: 5ʹ-CGC GCT TTT GTT TGG AGA GTT TGA TCC TGG-3ʹ and R: 5ʹ-GAG AAA GGA GAT CCA GCC GC-3ʹ. These primers were designed using the genetic program MTBC H37Rv ATCC 27294 with a 1537-bp specific region on the MTBC 16S rRNA gene as a target. The primers were added to the PCR Mix (KapaBiosystem^®^ Ready Mix).

#### PCR amplification

Amplification was initiated with denaturation at 94 °C for 20 s, followed by annealing at 53.8 °C for 10 s, and extension at 72 °C for 30 s [16]. The PCR results were visualized using electrophoresis followed by UV transillumination. The 16S rRNA gene of *M. tuberculosis* H37Rv strain ATCC 27294 was used as a positive control, and PCR mix (KapaBiosystem^®^ Ready Mix) without DNA was used as a negative control.

#### Gene Xpert

The Gene Xpert MTB/RIF test (Cepheid, CA, USA) was performed in the Department of Clinical Microbiology, Dr. Soetomo Academic Hospital in accordance with the standardized procedure provided in the accompanying manual [18].

#### Statistical analysis

The sensitivity and specificity of serial tests for TB detection were conducted by applying the culture method as the Gold Standard and performing *t*-tests in a 2 × 2 table analysis.

### Results

We obtained sputum samples from 96 pulmonary TB patients in Indonesia. These patients included 56 men (58.33%) and 40 women (41.67%). Most of the patients were aged around 30–60 years old (78.13%), and most of the patients had a drug treatment history of less than 6 months (89.6%).

All the samples were tested using AFB smears, the culture method, Gene Xpert assays, and nucleic acid amplification. The sensitivity of these serial tests was calculated based on the culture method as the Gold Standard. Samples with negative AFB smear results were often positively detected using a nucleic amplification test, with a sensitivity of 83.1%. The positivity result of PCR in negative AFB smear same with Gene Xpert (Table 1). On the other hand, negative culture result can be detected by PCR until 50 samples with specificity 79.17%. Furthermore, the amplification of nucleic acid similarly yielded positive results for samples that had negative culture results. These joint results were comparable to the Gene Xpert results.

**Table 1:** Results of serial tests from the tuberculosis patients

	PCR		Comparison
	Positive	Negative	
AFB			
Positive	*59*	20	Sensitivity: 83.1%
Negative	12	5	Specificity: 20%
*Gene Xpert*			
Positive	*59*	21	Sensitivity: 63.64%
Negative	12	4	Specificity: 16.67%
Culture			
Positive	22	5	Sensitivity: 30.56%
Negative	*50*	19	Specificity: 79.17%

Italic indicates the majority of samples

### Discussion

The AFB smear detects all acid-fast bacilli, including *Mycobacterium tuberculosis* and Non-Tuberculous Mycobacteria (NTM), it makes AFB smear not specific. Centrifugation and filtration can increase the concentration of sample to improve sensitivity of AFB smear [19], although there is AFB smear without concentration which is used and can cause many limitations. Additionally, this method still has low sensitivity for confirming MTBC infection [20]. Notably, a single AFB-positive sputum smear is now considered sufficient for a TB diagnosis [21, 22].

The culture method is necessary to confirm drug susceptibility, particularly for second-line drugs in cases of multi-drug resistance (i.e., MDR TB). The application of *M. tuberculosis* culture and phenotypic drug susceptibility testing (DST) requires significant training, infrastructure, strict infection control, and on-going quality assurance; however, in most countries, these are available in only regional reference laboratories. In this study, results based on only the culture method showed low positivity, which could be due to the requirements of this method for appropriate media, proper technique, and high quality specimens. Before the culture method was performed, the specimens were decontaminated, which required careful handling because a NaOH concentration that is too high will kill the bacteria but one that is too low will allow sample contamination [23]. Other study said culture is still as Gold standard for diagnosis of TB, but it is more difficult and may not be available, this method also required as much as 6–8 weeks to collect the result [24].

In the present study, they found 6.6% of rifampicin resistant strains (DST proven) revealing no mutations of rifampicin in the RRDR of *rpoB* gene in Gene Xpert assay, even if has a specific cartridge that was developed to simultaneously detect *M. tuberculosis* and determine its resistance to rifampicin [25, 26]. However, many of the patients diagnosed based on Gene Xpert assay results would also have been appropriately diagnosed based on chest X-ray findings or on clinical findings consistent with TB, and the extent to which the use of the Gene Xpert assay will increase the number of detected TB cases is not yet clear. Theoretical studies suggest that the application of this test will improve treatment targeting, with fewer patients who do not have TB incorrectly started on anti-TB treatment and a greater number of smear-negative “true TB” cases detected [27].

Nucleic acid amplification tests (NAAT), in which the most important one is the polymerase chain reaction (PCR), have emerged as powerful tools for rapid detection of the mycobacteria in clinical and research specimens [24]. The PCR method applied in this study used the 16S rRNA gene. Although both are molecular methods, this test is distinct from the method used by the Gene Xpert assay. Notably, the PCR method can identify cases of pulmonary TB infection from sputum samples more specifically compared with the other available methods [28]. The 16S rRNA gene is a universal gene in bacteria; this gene is functional and contains both a conserved region and a variable region [29]. Methods using this target gene allow the specific design of primers for the MTBC 16S rRNA gene; thus, only cases with MTBC infection yield positive results.

Some of the results obtained by other methods are not same as that by PCR because of the weakness of each method, so the serial test is important to determine and to distinguish the pulmonary TB infection from other diseases. Notably, it is best to apply all these methods as TB diagnostic tests before anti-TB drug treatment is begun [30]. In Indonesia, the diagnosis of new TB cases typically still occurs without the application of the Gene Xpert or PCR methods, although the Gene Xpert assay is used in Indonesia to confirm active TB in drug-resistant cases.

In conclusion, the application of serial tests as a new algorithm for MTBC detection can increase the validity of the TB diagnostic method, and this will better guide the management of TB treatment. The previous algorithm consists of AFB smear, culture method, and nucleic acid amplification test [31]. Our results provide a good comparison among methods, improve and indicate that the use of serial detection tests can identify cases that might otherwise fail to be detected.

## Limitations

This is the first study to investigate the molecular detection using a gene region that could be used to identify active TB cases but is insufficient for other uses, i.e., detecting the drug resistance of TB.

## Data Availability

The data set from the current study is available upon request from the corresponding author.

## Abbreviations

TB: tuberculosis
MTBC: *Mycobacterium tuberculosis* complex
AFB: acid fast bacilli
NTM: non-tuberculous Mycobacteria
LJ: Lowenstein–Jensen
PCR: polymerase chain reaction
DST: drug susceptibility testing
